# Implementing a National Approach to Research Ethics Review during a Pandemic – the Irish Experience

**DOI:** 10.12688/hrbopenres.13146.2

**Published:** 2020-11-16

**Authors:** Aileen Sheehy, Jennifer Ralph James, Mary Horgan

**Affiliations:** 1 National Office for Research Ethics Committees, Dublin, Ireland; 2 Royal College of Physicians of Ireland, Dublin, Ireland; 3 School of Medicine, University College Cork, Cork, Ireland

**Keywords:** Research Ethics, National Research Ethics Committees, Research Integrity, COVID-19

## Abstract

The surge of coronavirus disease 2019 (COVID-19) research studies involving human participants in response to the pandemic has meant that research ethics committees across the world have been challenged to adapt their processes to meet demand while retaining high standards of review. Ethics review during this pandemic remains essential to ensure the safety, dignity and well-being of research participants, however research ethics committees are now faced with new, and often complex, ethics considerations and logistical challenges.

This Open Letter looks specifically at the Irish experience of establishing a national approach to research ethics review amidst a global pandemic. This represents Ireland’s first National Research Ethics Committee, which provided the research community with an expedited and ‘single national opinion’ for ethics review for COVID-related research. The insights gleaned and lessons learned from the Irish experience may inform emergency responses to future pandemics or public health emergencies.

## Overview

In December 2019, a novel virus with pandemic potential was identified in Wuhan, China – severe acute respiratory syndrome coronavirus 2 (SARS-CoV-2), responsible for coronavirus disease 2019 (COVID-19) ^1^. The World Health Organization (WHO) declared COVID-19 a global pandemic on 11 March 2020 ^2^.

As with previous global pandemics, research was deemed to be essential in the global response to COVID-19 ^3^.

*‘The National Action Plan for Ireland’s Response to COVID-19’* included a clear objective to harness the capacity of the research and evidence community in Ireland to support immediate decision making during the pandemic ^4^. The implementation of a robust, expedited ethics review process was fundamental in enabling this research.

In accordance with the WHO’s ‘ *A Coordinated Global Research Roadmap*’ and on recommendation from the National Public Health Emergency Team (NPHET), the Irish Minister of Health established a temporary and dedicated national research ethics committee (NREC COVID-19) to deliver an expedited process for review of COVID-19-related health research ^3^.

A select committee of 19 members was appointed by the Minister to the NREC COVID-19 based on the appropriate diversity of expertise, skills, knowledge and perspectives to ensure the highest standards of ethics review. The NREC COVID-19 was operationalised and supported by the newly founded National Office for Research Ethics Committees, an independent office with a statutory function, housed with the Health Research Board.

In line with its terms of reference, the NREC COVID-19 was tasked with the ethics review of COVID-related ‘health research’ as defined within the Health Research Regulations 2018 ^5^. The Chair, in consultation with the National Office, refined the committee’s scope to meet both the evolving needs of the research community and the broader strategic national research agenda, prioritising review of studies that would most benefit from an expedited single national opinion.

## General challenges with ethics review during the COVID-19 pandemic

Ethics review is essential for maintaining high standards of research integrity, protecting participants in research and research workers from harm or exploitation, and providing reassurance to the public that these standards are being met ^6^. This is emphasised for pandemic situations where public trust is essential.

Research conducted during global health emergencies and pandemics raises particularly complex ethics challenges ^7^. This has been true for COVID-19 where the surge of new COVID-19 studies involving human participants has created a wide range of new ethics considerations and magnified others.

Existing ethics review systems and processes face demand for a prompt and efficient review process necessary to expedite essential research. The adaptation of ethics review processes is necessary to ensure timely review that maintains best practice ^8^.

Research practices have had to adapt during pandemics. This has meant that ethics committees are faced with novel, and often complex, ethics considerations ^9^. 

As strongly recommended during previous pandemics, research ethics committees (RECs) are encouraged to work closely with other regulatory and research bodies to ensure aligned and expedited approaches ^10^.

The need to build trust and engagement in new processes can often be overlooked in global health emergencies. Engagement and trust-building in the response to disasters like hurricane Katrina and the 2004 tsunami have led to long-term benefits. Where these have not been prioritised, often the needs of the communities involved have been neglected ^11^.

These challenges often need to be addressed with the backdrop of limited human and financial resources.

## International response to ethics review of research during the COVID pandemic

In March 2020, the WHO published ‘ *A Coordinated Global Research Roadmap: 2019 Novel Coronavirus*’ ^4^. This Roadmap reiterates that pandemics such as COVID-19 do not overrule the need to uphold ethical standards. It emphasises the need for countries to facilitate accelerated ethics review in emergency situations without compromising human participants’ protection.

The WHO also drafted guidance for RECs for rapid review of research during public health emergencies; this guidance covers the lifecycle of an ethics review application including electronic submission, efficient mobilising of committee expertise, virtual committee deliberations and prompt two-way communications between the committee and researchers ^8^.

On the back of these publications, many countries have created new, or adapted existing, processes to provide expediated ethics guidance and review of COVID-related research in the changing context of the pandemic.

The UK has a national research ethics review process through the Health Research Authority (HRA). During the COVID-19 pandemic, the HRA amended their processes to allow for a two tier fast-tracked ethics review process for COVID-related research ^12^. The first reviews ethics applications within 72 hours of submission and prioritises studies on vaccines, diagnostics, treatments, understanding of immune responses and disease prevalence. The second reviews studies within two weeks of submission and prioritises studies on the wider impact and general understanding of the COVID-19 pandemic.

The Netherland’s Central Committee on Research Involving Human Subjects have adapted their processes to expedite ethics review of COVID-related research ^13^. This adapted process prioritises vaccines but can be used for other intervention studies. The Committee meet within 7 days of submission of the ethics application and the total duration of the review was a maximum of 25 days.

The European Network of Research Ethics Committees released a position paper on ‘ *The Responsibility of Research Ethics Committees during the COVID-19 Pandemic*’ ^14^. This paper clearly outlines the prioritisation of studies focussed on the prevention or treatment of COVID-19, the need for adapted processes for ethics review and the maintained importance of informed consent of participants in COVID-related research.

The European Medicines Agency published ‘ *Guidance on The Management Of Clinical Trials During The Covid-19 (Coronavirus) Pandemic*’, which includes guidance for researchers to ensure trials of medicinal products are safe and ethical ^15^.

## Irish response to ethics review during the COVID-19 pandemic

Prior to the pandemic, Ireland’s ethics review system was restricted to several dozen (estimates of up to 80) RECs operating at a local or institutional level. This is largely regarded as disjointed and inefficient ^16^. Twelve RECs are formally recognised by the Department of Health to provide single national opinions for Clinical Trials of Investigational Medicinal Products.

In July 2019, the Department of Health published the ‘ *General Scheme of the National Research Ethics Committees Bill*’ offering the research community an insight into what nationalising research ethics review may look like ^17^. Irish legislation based on this Bill will modernise the current system of ethics review with a streamlined, regulated and fit-for-purpose national system.

With COVID-19 case numbers increasing across Ireland in March 2020, the existing ethics review structures were not equipped to deliver the accelerated and unified approach needed for COVID-related research. A national approach to ethics review for COVID-related research was clearly required in Ireland.

Concurrently, the National Office for Research Ethics Committees was established with a priority remit to develop a national ethics review structure to meet the requirements of the cross-European Clinical Trials Regulation (EU) No 536/2014, due to come into effect in 2021 ^18^.

The Minister for Health established a temporary National Research Ethics Committee for COVID-19 (NREC COVID-19) – Ireland’s first National Research Ethics Committee. Members were selected based on their diverse knowledge, expertise, and previous experience on local RECs, with due regard for the diversity of skills and experience necessary to enable an informed review of the applications likely to come under review in line with international best practice. The NREC COVID-19 benefitted particularly from the contributions of those members representative of Public and Patient Involvement.

This National Committee provided the research community with an expedited and single national ethics opinion for COVID-related health research. The newly established National Office for Research Ethics Committees was tasked with all aspects of operationalising and supporting the NREC COVID-19. Some local RECs continued to operate throughout the pandemic providing an agile ethics review process for both local COVID and non-COVID research projects.

The work of the NREC COVID-19 was informed by national guidance contained in the Ethical Framework for Decision-making in a Pandemic, which makes particular reference to the values of fairness, reciprocity and privacy as guiding values for research during a pandemic ^19^. The NREC COVID-19 was also informed by international guidance from the World Health Organization, *‘Ethical standards for research during public health emergencies: distilling existing guidance to support COVID-19’* ^20^.

Given the government’s recommendations on social distancing and travel restrictions, the committee was formed virtually and was dependent on technology to discharge its duties.

For the initial three-month tenure of the NREC COVID-19, the committee met weekly. Decisions were made by consensus and a quorum was ensured at each meeting. Members dedicated at least four hours per week between reviewing applications and participating in the meeting.

Due to ongoing demand for a single national opinion for COVID-related studies, the initial three-month tenure was extended by an additional seven weeks and the NREC COVID-19 met three times during this period.

From the outset, the NREC COVID-19 aligned its approach with other national regulatory bodies to ensure a coordinated approach to accelerate health research. The application process was merged with that of the Health Research Consent Declaration Committee (HRCDC), a national statutory committee that grants consent declarations, or waivers, for health research studies where obtaining explicit consent is not feasible. Both the NREC COVID-19 and the HRCDC ran their processes in parallel to ensure robust, accelerated and coordinated review processes ^5^. The NREC COVID-19 maintained close contact with the Health Products Regulatory Authority (HPRA), the national competent authority for clinical trials and medical devices in Ireland, with a view to ensuring consistency with regulatory review processes.

Over its tenure, the NREC COVID-19 reviewed 93 applications. Of the 93 applications reviewed, four were declined, 83 provisional approvals with requests for clarifications or conditions set, and 85 final approvals. Studies included basic scientific and social research, clinical trials, epidemiology research and applied research. The NREC COVID-19 approved research that will be carried out within 61 institutions across 20 out of the 26 counties in the Republic of Ireland. A total of 23 studies approved by the NREC COVID-19 were part of international collaborations.

The often-complex ethical considerations faced by the NREC COVID-19 were primarily COVID-specific or particular to a public health emergency. These included best practices capturing informed assent for incapacitated patients, changes to consent models due to social restriction guidelines and ethical and data protection considerations for new digital communication and research platforms. Frequent areas of feedback from the Committee to applicants included clarifications around data protection and issues around clarity and readability of patient information leaflets and consent forms.

Anecdotal feedback from Committee Members found that the key motivations to participate in the NREC COVID-19 were to contribute to the national response, to enable Irish researchers and to ensure high standards of ethics review during COVID-19.

## Challenges and barriers – how they were overcome

As the NREC COVID-19 was the first National Research Ethics Committee in Ireland and was rapidly convened during a pandemic, a number of challenges arose during initial set-up and over the tenure of the committee.

Establishing a new ethics review system during a pandemic presented several logistical challenges, largely due to restrictions on face-to-face meetings, travel and consultations. At the development stage, stakeholders from the other regulatory agencies, convened by the Department of Health, held regular conference calls to delineate this new ethics reviews process in an informed and coordinated manner. Furthermore, communication was established with national research funders, including the Health Research Board (HRB) and Science Foundation Ireland (SFI), to inform temporal prediction of application volumes.

At the beginning of their tenure, Committee Members received IT training in both the selected video conferencing facility and use of an online digital reading room to ensure that remote meetings ran smoothly and securely.

Interest from the research community in the NREC COVID-19 was high as evidenced by application submissions. Accordingly, early into the NREC COVID-19’s tenure, the Committee’s scope was refined to prioritise study types for review. The studies prioritised by the NREC COVID-19 included clinical trials, multi-centred studies, national and international studies, and data linkage studies.

As researchers themselves had to adapt to requisite changes to research practices due to COVID-19, the NREC COVID-19 was faced with complex ethics considerations. The NREC COVID-19 endeavoured to provide consistent feedback to the research community and discussed its shared alignment on complex issues with other regulatory bodies.

As both the National Office and the NREC COVID-19 were new additions to the research infrastructure, it was essential to engender the trust and confidence of the research community and the wider public. The implementation of transparent processes such as a dedicated webpage, frequently asked questions (FAQs) and timely publication of meeting minutes and decisions assisted in this regard.

The National Office is hosted as an independent statutory office by the HRB. The National Office leveraged the HRB’s support for promotion and proactive communication to the research community. This fostered understanding of the remit of the National Office and the NREC COVID-19.

Entrenching a single national opinion for ethics review in the research environment was novel in Ireland. It was therefore essential that roles between the local RECs and the NREC COVID-19 were clearly delineated and two-way communication established. Local RECs were provided with weekly summaries of decisions from the NREC COVID-19 and regular general updates provided by the National Office. A local REC manager agreed to act in a liaison capacity between the NREC COVID-19 and local RECs.

## What can be learned from Irish experience?

The time and resources invested to thoroughly design expedited processes and timelines informed by other regulatory processes ahead of the launch of the Committee was an effective and worthwhile exercise. All valid and complete applications that were submitted ahead of the weekly submission deadlines were reviewed within 7 days of the deadline. All 93 applications reviewed received a decision letter generally within one to two days of a NREC COVID-19 meeting.

The coordinated approach and open communication channels across several regulatory bodies for health research from the outset means that strong partnerships have been made for future endeavours. The mutual objective of accelerating Irish health research despite the unprecedented environment ensured momentum was maintained throughout. The shared learnings and alignments on complex issues can now feed into improved research practices and research integrity nationally.

The firm commitment to transparency during the term of the NREC COVID-19 has been widely recognised as a positive move to encourage trust, openness and integrity in the research process

A key learning was the need for a targeted scope from the outset of the Committee’s work. Refining the scope during the NREC COVID-19 tenure led to some confusion in the research community and the local RECs. However, the agility of the Committee to adapt to the needs of the research environment is testament to the process. A clearly defined scope of research for prioritised review from the outset of a National REC in response to a pandemic will streamline processes and reduce uncertainty.

Due to time restraints and the necessity to reflect multiple review processes in one form, the application form was perceived by some in the research community to be dense. An online application system would have benefited this streamlined process. If time allowed, a more user-friendly approach would be to tailor ethics application forms to specific COVID-related study types. Centralised portals being developed at a European level in response to the European Clinical Trial Regulation (EU 536/2014) and the Medical Device Regulation (EU 2017/745) will help support a more streamlined and standardised approach to research ethics across Europe in these areas of research.

Notwithstanding the benefits of technology, the successful operation of NREC COVID-19 relied both on the commitment and efficiency of the Chair and Committee Members and the dedicated staff at the National Office.

## Conclusion

The NREC COVID-19 played a significant role in Ireland’s research response to COVID-19, accelerating research locally and enabling Ireland’s participation in forefront research internationally, thereby supporting Irish contribution to the global research effort.

Now more than ever, it is imperative that countries learn from each other’s responses to the global pandemic. Much can be learned from the Irish experience of setting up a national system for research ethics review during a global pandemic.

Implementation of the NREC COVID-19 demonstrates what can be achieved with cross-agency coordination, dedicated resources and individuals’ commitment under a unified vision; moreover it is proof of principle for a national strategic approach to research ethics review and gives credence to pending Irish legislation on foot of the National Research Ethics Committees Bill.

## Data availability

### Underlying data

No data are associated with this article
