# Supplementary material for: Implementing a National Approach to Research Ethics Review during a Pandemic – the Irish Experience
Source: HRB Open Res. 2020 Nov 16;3:63. Originally published 2020 Sep 11. [Version 2] doi: 10.12688/hrbopenres.13146.2 (PMC7797935; doi:10.12688/hrbopenres.13146.2)
Supplement: Peer review history

# Peer review: Reviewer response for version 2

**DOI:** 10.21956/hrbopenres.14313.r28373

**Published:** 2021-01-08

**Authors:** Joop van Gerven, Johannes J. M. van Delden

**Affiliations:** 1 Central Committee for Research involving Human Subjects (CCMO), The Hague, The Netherlands; 2 Leiden University Medical Center, Leiden, The Netherlands; 3 Centre for Human Drug Research, Leiden, The Netherlands; 4 Department of Public Health, Healthcare Innovation & Evaluation and Medical Humanities (PHM), Julius Center Research Program Methodology, University Medical Center Utrecht, Utrecht, The Netherlands; 5 Julius Center for Health Sciences and Primary Care, Medical Humanities, University Medical Center Utrecht, Utrecht, The Netherlands

The authors have provided extensive and insightful responses to our questions. Their additions to the paper are adequate. The letter is a useful contribution to discussions on medical ethics regulations.

Does the article adequately reference differing views and opinions?

Yes

Are all factual statements correct, and are statements and arguments made adequately supported by citations?

Yes

Is the Open Letter written in accessible language?

Yes

Where applicable, are recommendations and next steps explained clearly for others to follow?

Partly

Is the rationale for the Open Letter provided in sufficient detail?

Partly

Reviewer Expertise:

JvG: research governance, clinical pharmacology, neurology; HvD: medical ethics

We confirm that we have read this submission and believe that we have an appropriate level of expertise to confirm that it is of an acceptable scientific standard.

# Peer review: Reviewer response for version 2

**DOI:** 10.21956/hrbopenres.14313.r28372

**Published:** 2020-11-17

**Authors:** Rachel K. Crowley

**Affiliations:** 1 Department of Endocrinology, St Vincent's University Hospital, Dublin, Ireland; 2 University College Dublin, Dublin, Ireland

Thank you for addressing the points made in the review, and contributing to the literature on the practicalities of delivering robust ethical review.

 I suggest the Authors make the approval numbers more clear in the Irish Response section, paragraph 12. Otherwise happy to approve.

Does the article adequately reference differing views and opinions?

Partly

Are all factual statements correct, and are statements and arguments made adequately supported by citations?

Yes

Is the Open Letter written in accessible language?

Yes

Where applicable, are recommendations and next steps explained clearly for others to follow?

Partly

Is the rationale for the Open Letter provided in sufficient detail?

Yes

Reviewer Expertise:

Endocrinology. Research Governance.

I confirm that I have read this submission and believe that I have an appropriate level of expertise to confirm that it is of an acceptable scientific standard.

# Peer review: Reviewer response for version 1

**DOI:** 10.21956/hrbopenres.14260.r27946

**Published:** 2020-10-06

**Authors:** Joop van Gerven, Johannes J. M. van Delden

**Affiliations:** 1 Central Committee for Research involving Human Subjects (CCMO), The Hague, The Netherlands; 2 Leiden University Medical Center, Leiden, The Netherlands; 3 Centre for Human Drug Research, Leiden, The Netherlands; 4 Department of Public Health, Healthcare Innovation & Evaluation and Medical Humanities (PHM), Julius Center Research Program Methodology, University Medical Center Utrecht, Utrecht, The Netherlands; 5 Julius Center for Health Sciences and Primary Care, Medical Humanities, University Medical Center Utrecht, Utrecht, The Netherlands

The urgent need for effective vaccines and treatments for COVID-19 has stimulated many organizations involved in research, development and regulatory approval to improve the efficiency of their processes. Ethics review is an essential part of clinical research, but it is inefficient to perform separate institutional or regional reviews for each participating research centre (as is current practice in most countries). This open letter discusses Ireland’s experience or implementing a National Research Ethics Committee for COVID-19 related research in humans, the NREC-COVID-19. It comes to the conclusion (P5-6/8) that *‘Much can be learned from the Irish experience of setting up a national system for research ethics review during a global pandemic.’* This reviewer agrees, and in this sense the letter is a welcome contribution to the improvement and harmonization of research ethics review processes in other (European) countries. The letter presents some background information, but largely focusses on organizational and procedural aspects of the implementation and operation of the NREC COVID-19. However, because of the relevance of Ireland’s experience for other countries, more insights and details would be needed than are offered in this letter. It may not be possible to address all the questions below in this open letter. But then perhaps they can help raise awareness for some remaining issues, that are important for the harmonization and integration of ethics reviews in drug and regulatory research and development.

*P.3/8: ‘A select committee of 19 members was appointed… based on the appropriate diversity of expertise, skills, knowledge and perspectives to ensure the highest standards of ethics review’. *

- What were the disciplines considered ‘to ensure the highest standards of ethics review’?
- Undoubtedly one or more lay persons were selected, but their experience with a novel disease must have been limited and/or very personal. There was (is?) also no patient association to be involved. During the course of the pandemic, the impact and consequences of the disease became more apparent. (How) did the NREC COVID-19 take this increasingly relevant patient perspective into consideration?

*P5/8: ‘it was essential to engender the trust and confidence of the research community and the wider public' *

- This part of the main text of the letter (and others) emphasizes the importance of public trust and transparency (eg *. *please also consider adding this important aspect to the Overview on P.3/8.
- How open and transparent was the selection process for committee members?

*P.3/8: ‘the NREC COVID-19 was tasked with the ethics review of COVID-related ‘health research’’*

- Many investigators will have felt the need to adapt their research to the limitations and possibilities offered by the COVID19-situation. This could have led to proposals which may have been interesting in their own right, but were only superficially related to COVID19. Was any specific framework or definition provided for research applications to NREC COVID-19? What happened to proposals that seemed unsuitable in this regard? Were they evaluated by the NREC COVID-19 and rejected, or sent back to local or regional committees by support staff form the National Office?
- According to Ireland’s National Action Plan (reference 4), a ‘bioethics subgroup’ was to be set up. Was this subgroup the precursor of NREC COVID19? This would suggests a focus on ‘bioethics’, but COVID19 poses a wide range of ethical problems for society (allocation of sparse resources (financial, personnel, health care), impact and balance of costs and benefits of public measures for individuals and society, etc). As indicated by the authors, many of these problems also directly affect clinical research *(P.3/8: ‘Research practices have had to adapt…’)*. Was any specific consideration given to this broader perspective during the composition of the NREC COVID-19 or its procedures (selection of disciplines and committee members, required information from investigators, standard items for agenda and discussions, public communication and transparency, etc)? Did any general learning points come up regarding ethics review of research that is under great public pressure?

*P.3/8: ‘As strongly recommended during previous pandemics, research ethics committees (RECs) are encouraged to work closely with other regulatory and research bodies to ensure aligned and expedited approaches.’ P.4/8 ‘the NREC COVID-19 aligned its approach with other national regulatory bodies to ensure a coordinated approach to accelerate health research.’*

- The authors draw attention to the importance of coordination with other regulatory and research bodies, but no further information is presented. This has undoubtedly been complex, but without going into detail, it would be informative for readers to have some insight into how this was organized and which problems were encountered. Did this ‘alignment’ also involve the medical universities or research institutes?)

*P.4/8: ‘Of the 93 applications reviewed, four were declined, 83 provisional approvals with requests for clarifications or conditions set, and 81 final approvals.’*

- Were any questions or grounds for rejection found to be specific for COVID-19? Or was this perhaps the case for any frequently recurring questions? Were the question mainly related to unfavourable risk/benefit balance, scientific shortcomings, undue concessions to the urgency of the situation, or health care limitations?

*P.5/8: ‘Furthermore, communication was established with national research funders (…) to inform temporal prediction of application volumes.’*

- Was the number of actual applications in line with these predictions? Or did most protocols come from other funders or sponsors? Did sponsorship (public vs private) influence prioritization (if not in principle, then perhaps in practice)?

*P.5/8 ‘the NREC COVID-19 was faced with complex ethics considerations.’*

- What were these ‘complex ethics considerations’?

*P5/8: ‘It was (…) essential that roles between the local RECs and the NREC COVID-19 were clearly delineated and two-way communication established.’ *

- How was this ‘two-way communication’ perceived by the regional or institutional review boards? Did these boards contribute to the deliberations of the NREC COVID-19?
- Many local or institutional of local RECs are closely associated with medical schools or research institutes, where RECs often also take the local situation and feasibility into consideration. How did the institutes or the researchers respond to this centralization of review responsibilities? Did the institutional review boards concerned about their loss of autonomy?
- Was (is) there any possibility for appeal?

*P.5/8: ‘A key learning was the need for a targeted scope from the outset of the Committee’s work. Refining the scope during the NREC COVID-19 tenure led to some confusion in the research community and the local RECs. However, the agility of the Committee to adapt to the needs of the research environment is testament to the process. A clearly defined scope of research for prioritised review from the outset of a National REC in response to a pandemic will streamline processes and reduce uncertainty.’*

- It is a bit unclear what is meant by ‘ *needs of the research environment*’ here (investigators? health care system? society? patients?), but it seems that the authors expect a National Research Ethics Committee to be focused, but also flexible. They acknowledge that this may generate confusion among researchers and local or regional ethics committees. Considering the consequences, was any structure in place to involve the research and ethics review community in the decision to centralize reviews on a national level, that matched the required ‘agility’?

*P5/8: ‘An online application system would have benefited this streamlined process.’*

- Such systems (‘portals’) are currently being developed for the European Clinical Trial Regulation (EU 2014/536) and the Medical Device Regulation (EU 2017/745). It remains to be seen whether these portals will be perceived by researchers to be less ‘dense’, but for many applications they will be inescapable and it would be useful to mention them.

*P.5/8: ‘the successful operation of NREC COVID-19 relied (also) on (…) the dedicated staff at the National Office’*

- Dedication is an important asset, but to which extent did the requirements for supportive staff also include scientific expertise? Was staff also involved in the content of research applications, and if so – how important was this for the quality, speed and harmonization of the review process and for the accommodation of committee members?

 

 Some general questions:

- Could something be shared about the investment in the setup of the NREC COVID-19? How much time and effort (shareholder meetings) did this take? How much friction can be expected when the perceived public need for centralization is less pressing?
- Were important legal changes in (other) national laws or regulations necessary for the setup of the NREC COVID-19 (or perhaps foreseen for a future more general NREC)?
- The ‘learning experiences’ seems to have been positive, and few mistakes or miscalculations seem to have been made. This is commendable, but would in hindsight perhaps other decisions have been made that help other countries?
- How did (or will) the experience with the NREC COVID-19 influence ideas about the design of a centralized ethics and regulatory review system in the future (in Ireland, but perhaps also in a European perspective, considering the increasing influence of European regulations like European Clinical Trial Regulation (EU 536/2014), Medical Device Regulation (EU 2017/745) and In Vitro Diagnostics Regulation (EU 2017/746))?
- (It probably remains to be seen which scope will be chosen for a ‘general NREC’a in the future, outside of an epidemic. The Central Review Decree of the Dutch Central Committee on Research Involving Human Subjects (CCMO) could serve as an example ( https://english.ccmo.nl/investigators/legal-framework-for-medical-scientific-research/decrees-and-ministerial-regulations/central-review-of-medical-research-involving-human-subjects-decree-bcb).)

Does the article adequately reference differing views and opinions?

Yes

Are all factual statements correct, and are statements and arguments made adequately supported by citations?

Yes

Is the Open Letter written in accessible language?

Yes

Where applicable, are recommendations and next steps explained clearly for others to follow?

Partly

Is the rationale for the Open Letter provided in sufficient detail?

Partly

Reviewer Expertise:

JvG: research governance, clinical pharmacology, neurology; HvD: medical ethics

We confirm that we have read this submission and believe that we have an appropriate level of expertise to confirm that it is of an acceptable scientific standard, however we have significant reservations, as outlined above.

# Response

**Published:** 2020-11-03

**Authors:** Aileen Sheehy

**Affiliations:** Health Research Board, Ireland

Dear Prof van Gerven, 

 Thank you for taking time to review our work, which will help us improve this Open Letter. We have revised the publication, informed by your comments and those of the second reviewer. We have also replied to your comments and queries directly below:

- *P.3/8: ‘A select committee of 19 members was appointed… based on the appropriate diversity of expertise, skills, knowledge and perspectives to ensure the highest standards of ethics review’.*

*What were the disciplines considered ‘to ensure the highest standards of ethics review’?*

*Undoubtedly one or more lay persons were selected, but their experience with a novel disease must have been limited and/or very personal. There was (is?) also no patient association to be involved. During the course of the pandemic, the impact and consequences of the disease became more apparent. (How) did the NREC COVID-19 take this increasingly relevant patient perspective into consideration?*

 Members of the NREC COVID-19 were formally appointed by the Minister of Health. Members were selected based on their knowledge, expertise, and previous experience on local research ethics committees (RECs), with due regard for the diversity of skills and experience necessary to enable an informed review of the applications likely to come under review In line with international best practice.

 As the NREC COVID-19 was set up as a matter of urgency, time did not allow for formal training of members in research ethics. For this reason, many of the committee members were selected based on their experience sitting on RECs or similar types of committees. The NREC was very mindful of existing trusted ethics resources including the cornerstone of ethics review, the Declaration of Helsinki, and the WHO ‘Guidance for research ethics committees for rapid review of research during public health emergencies’.

 As you mention, the Committee was established in the earlier stages of the pandemic in Ireland. As far as we are aware, there were no patient associations directly representing patients recovering from, or impacted by, COVID-19 in Ireland at that time. However, the areas of expertise within the NREC COVID-19 were very relevant to the types of complex ethical considerations that can arise from COVID-related research. Expertise included infectious diseases, medical research, frontline healthcare worker experience, clinical trials, statistics, consent and decision-making capacity, patient association and patient/public advocacy. The NREC COVID-19 benefitted particularly from the contributions of the public and patient representatives.

- *P5/8: ‘it was essential to engender the trust and confidence of the research community and the wider a*

*This part of the main text of the letter (and others) emphasizes the importance of public trust and transparency (eg. please also consider adding this important aspect to the Overview on P.3/8.*

*How open and transparent was the selection process for committee members?*

 As the Committee was set up as a matter of urgency, members were selected based on their substantial and diverse expertise, and many necessarily had prior REC experience. The details of the NREC COVID-19 membership are published openly along with the minutes from all the Committee meetings. Senior National Office staff sat in on meetings as observers and can attest to the rigour of the review deliberations.

- *P.3/8: ‘the NREC COVID-19 was tasked with the ethics review of COVID-related ‘health research’’*

*Many investigators will have felt the need to adapt their research to the limitations and possibilities offered by the COVID19-situation. This could have led to proposals which may have been interesting in their own right, but were only superficially related to COVID19. Was any specific framework or definition provided for research applications to NREC COVID-19? What happened to proposals that seemed unsuitable in this regard? Were they evaluated by the NREC COVID-19 and rejected, or sent back to local or regional committees by support staff form the National Office?*

 Referring to the section ‘Challenges and barriers – how they were overcome’, we mention briefly that the scope of research reviewed by the NREC COVID-19 was refined early in the process to mitigate this problem. For workflow management purposes and Committee capacity, it was necessary to prioritise the scope of research for their review.

 Several applications were submitted to the NREC COVID-19 that did not fit within the prioritised scope. For these studies, the National Office suggested that applicants may be better served using their local REC; in most instances, this was the option chosen by the applicant. In a few instances, the applicant put forward strong justification why their study should be reviewed by the NREC COVID-19 rather than a local REC. For these, the Chair decided whether the applications would be reviewed by the NREC COVID-19.

- *According to Ireland’s National Action Plan (reference 4), a ‘bioethics subgroup’ was to be set up. Was this subgroup the precursor of NREC COVID19? This would suggests a focus on ‘bioethics’, but COVID19 poses a wide range of ethical problems for society (allocation of sparse resources (financial, personnel, health care), impact and balance of costs and benefits of public measures for individuals and society, etc). As indicated by the authors, many of these problems also directly affect clinical research  (P.3/8: ‘Research practices have had to adapt…’). Was any specific consideration given to this broader perspective during the composition of the NREC COVID-19 or its procedures (selection of disciplines and committee members, required information from investigators, standard items for agenda and discussions, public communication and transparency, etc)? Did any general learning points come up regarding ethics review of research that is under great public pressure?*

- The Bioethics sub-group (now called the Pandemic Ethics Advisory Group) and the NREC COVID-19 are separate, independent entities. The NREC COVID-19 was solely responsible for the ethics review of COVID-related research involving human participants. The Bioethics subgroup looked at broader ethical issues, beyond research, in relation to Ireland’s national response to COVID-19. They are a subgroup of the National Public Health Emergency Team, which provides recommendations to the Irish government to inform the national response to the plethora of health and socioeconomic issues that are arising from that pandemic.

 - The composition of the NREC COVID-19 reflected the diversity of expertise that was likely required to assess the ethics of COVID-related research involving human participants. Discussion topics such as sparse resources including limited-supply medicines did come up in relation to COVID-19 research and clarifications from applicants were sought when needed. The NREC COVID-19 drew from guidance laid out in the Pandemic Ethics Advisory Group’s publication, *Ethical Framework for Decision-making in a Pandemic*, which speaks to issues such as allocation of scarce resources.

 - The area of capacity and consent was a frequent discussion point and is an area of particular complexity in Ireland given evolving guidance on this topic. In the interests of consistency and clarity, the NREC COVID-19 and the National Office developed a briefing document describing the Committee’s aligned position on capacity and consent. This was then shared with the local RECs, who were welcoming of this information.

- *P.3/8: ‘As strongly recommended during previous pandemics, research ethics committees (RECs) are encouraged to work closely with other regulatory and research bodies to ensure aligned and expedited approaches.’ P.4/8 ‘the NREC COVID-19 aligned its approach with other national regulatory bodies to ensure a coordinated approach to accelerate health research.’*

*The authors draw attention to the importance of coordination with other regulatory and research bodies, but no further information is presented. This has undoubtedly been complex, but without going into detail, it would be informative for readers to have some insight into how this was organized and which problems were encountered. Did this ‘alignment’ also involve the medical universities or research institutes?)*

 We provide more detail of our integrated coordination with two key health research regulatory bodies in Ireland – the Health Products Regulatory Agency and the Health Research Consent Declaration Committee – in the section ‘Irish response to ethics review during the COVID-19 pandemic’.

 As many of the local RECs are based in medical universities and research institutions, we maintained close contact with these institutions through the local RECs, in addition to direct communication with the VPs of Research on occasion. See section ‘Challenges and barriers – how they were overcome’ for more information on this.

- *P.4/8: ‘Of the 93 applications reviewed, four were declined, 83 provisional approvals with requests for clarifications or conditions set, and 81 final approvals.’*

*Were any questions or grounds for rejection found to be specific for COVID-19? Or was this perhaps the case for any frequently recurring questions? Were the question mainly related to unfavourable risk/benefit balance, scientific shortcomings, undue concessions to the urgency of the situation, or health care limitations?*

 One application was declined based on the unfeasibility of the project’s methodology due specifically to COVID-19. However, the applicant revised the methodology in light of the Committee’s response and the study was later approved.

 There were frequently occurring clarifications requested by the NREC COVID-19 as part of the decisions made. These included clarifications around data protection, improvements to the readability of Patient Information Leaflets, and improvements to consent forms.

- *P.5/8: ‘Furthermore, communication was established with national research funders (…) to inform temporal prediction of application volumes.’*

*Was the number of actual applications in line with these predictions? Or did most protocols come from other funders or sponsors? Did sponsorship (public vs private) influence prioritization (if not in principle, then perhaps in practice)?*

 The peak workflow for NREC activity somewhat aligned with the outcomes of the national funding calls. It emerged that many of the studies assessed by the NREC COVID-19 were from applicants based in healthcare settings. In these instances, although the applicants had permission to undertake the research at the proposed healthcare settings, the studies were not affiliated with any funding agency or private sponsors, and no specified funding required.

 Prioritisation was never based on sponsorship type.

- *P.5/8 ‘the NREC COVID-19 was faced with complex ethics considerations.’*

*What were these ‘complex ethics considerations’?*

 Although there were many new challenges setting up a national REC process, the emphasis of the complexity lay in the ethical considerations, which were primarily COVID-specific or particular to a public health emergency. Many prospective participants for COVID research studies may be incapacitated and a consent declaration required; consequently, one complex consideration was the justification for, and best practice in, capturing informed assent from people close to the participant such as family members, partners or friends.

 In terms of enabling informed consent, e-consent and telephone consent was used frequently in the research studies under review due to the social restrictions and guidance in place. The potential implications for these approaches were discussed by the Committee.

 Due to the prevailing social restrictions and guidance, many new digital communication and research platforms were used in COVID-related studies. The implications of using these platforms for the protection of personal data of participants was another complex consideration. While mindful of the imperative to share knowledge in a timely manner, the Committee frequently sought evidence and reassurances that participants’ personal information would be protected throughout the research study when managed by any digital tools, including within international collaborations.

- *P5/8: ‘It was (…) essential that roles between the local RECs and the NREC COVID-19 were clearly delineated and two-way communication established.’*

*How was this ‘two-way communication’ perceived by the regional or institutional review boards? Did these boards contribute to the deliberations of the NREC COVID-19?*

 The National Office proactively communicated (by email) to the local RECs all key developments with the national system and all decisions made, in addition to the Head attending a meeting of the local REC representatives. The National Office was open to reciprocal communication from the local RECs, and this was supported by a local REC manager who agreed at the invitation of the National Office to act in a liaison capacity between the NREC and the local RECs. Informal email and verbal communications from several RECs show that the work of the NREC and the National Office is welcomed. The local RECs did not contribute to NREC COVID-19 decisions, however their feedback on improving specific aspects of the process was considered. Importantly, the NREC COVID-19 respected decisions made at a local level and the local RECs respected decisions made by the NREC COVID-19 at a national level.

- *Many local or institutional of local RECs are closely associated with medical schools or research institutes, where RECs often also take the local situation and feasibility into consideration. How did the institutes or the researchers respond to this centralization of review responsibilities? Did the institutional review boards concerned about their loss of autonomy?*

Although the NREC COVID-19 was the first National Research Ethics Committee in Ireland, the concept of nationalising research ethics in Ireland has been in discussion for some time. Last year the General Scheme of the National Research Ethics Committees Bill was published as an important first step to shape what the national system will look like. It is expected that the associated legislation will be enacted in 2021.

 Both institutions and researchers were largely appreciative of the NREC COVID-19 initiative. For researchers, an expedited single national ethics opinion was welcome for their COVID-related research, which needed to be established quickly. For local institutions, the initiative alleviated many of the pressures of the high volume of COVID-19 research projects being undertaken at a time when some local RECs were not functioning.

 The addition of the NREC COVID-19 to the research infrastructure did uncover a number of existing ‘blind-spots’ in the research system, including oversight by some researchers to inform their local REC / Research Office of their research study. This and other opportunities for improvement in research governance are being considered by a local REC reform working group, on which there is National Office representation.

  

- *Was (is) there any possibility for appeal?*

There was no appeals Committee in place for decisions made by the NREC COVID-19, however applicants could resubmit a study addressing the prior concerns of the NREC COVID-19. An appeals process will be a component of the national system implemented through legislation on foot of the NREC Bill.

- *P.5/8: ‘A key learning was the need for a targeted scope from the outset of the Committee’s work. Refining the scope during the NREC COVID-19 tenure led to some confusion in the research community and the local RECs. However, the agility of the Committee to adapt to the needs of the research environment is testament to the process. A clearly defined scope of research for prioritised review from the outset of a National REC in response to a pandemic will streamline processes and reduce uncertainty.’*

*It is a bit unclear what is meant by ‘needs of the research environment’ here (investigators? health care system? society? patients?), but it seems that the authors expect a National Research Ethics Committee to be focused, but also flexible. They acknowledge that this may generate confusion among researchers and local or regional ethics committees. Considering the consequences, was any structure in place to involve the research and ethics review community in the decision to centralize reviews on a national level, that matched the required ‘agility’?*

 The research environment refers to all of the above – researchers, research institutions, healthcare systems and settings, patients and society at large.

 Although a National Research Ethics Committee should have a focused remit of research for review, they should remain flexible to an extent in how they work – this was key to dealing with COVID-19 research ethics review. One critical factor to consider was the unknown volume of research studies that would be prompted by the pandemic, and therefore the unknown workflow for the Committee. Many of the local RECs continued to work very flexibly during the pandemic (and continue to do so) enhancing the agility of the research system.

 As mentioned previously, discussions around nationalising research ethics continue to take place in Ireland and these discussions involve the research and ethics review communities.

- *P5/8: ‘An online application system would have benefited this streamlined process.’*

*Such systems (‘portals’) are currently being developed for the European Clinical Trial Regulation (EU 2014/536) and the Medical Device Regulation (EU 2017/745). It remains to be seen whether these portals will be perceived by researchers to be less ‘dense’, but for many applications they will be inescapable and it would be useful to mention them.*

 Agree and we will add them to the paper.

- *P.5/8: ‘the successful operation of NREC COVID-19 relied (also) on (…) the dedicated staff at the National Office’*

*Dedication is an important asset, but to which extent did the requirements for supportive staff also include scientific expertise? Was staff also involved in the content of research applications, and if so – how important was this for the quality, speed and harmonization of the review process and for the accommodation of committee members?*

 The National Office is staffed by two senior scientific staff. As part of the application validation process, staff did provide advice and guidance on occasion to researchers on their applications in advance of consideration by the NREC COVID-19, where there were clear administrative improvements to be made or where the application would benefit from additional supporting information. This took considerable time, in addition to operationalising Committee meetings, but overall the approachability of the National Office in this regard helped the NREC COVID-19 review run more efficiently and assisted researchers to strengthen their applications.

  

- *Could something be shared about the investment in the setup of the NREC COVID-19? How much time and effort (shareholder meetings) did this take? How much friction can be expected when the perceived public need for centralization is less pressing?*

The set-up of the NREC COVID-19 required the dedicated focus and time of several professional staff from the Department of Health, the Secretariat of the HRCDC and the HPRA. The implementation and ongoing operationalisation of the Committee was managed by two full-time senior staff and an administrative assistant at the National Office; this dedicated resourcing was a key enabler for the NREC COVID-19 and a factor that the local REC system doesn’t always benefit from.

 At an estimate the NREC COVID-19 Members dedicated at least four hours of their time each week – two hours for the Committee meeting and two or more hours of review ahead of the meeting, largely over weekends and outside of business hours. Due to the nature of their roles, the Chair and Vice-Chairs committed additional time.  We will add some information on this to the paper.

  As many strong relationships have now been formed during this intense period, it creates a strong foundation for the establishment of additional National RECs. We expect that this will reduce any actual or perceived friction.

  

- *Were important legal changes in (other) national laws or regulations necessary for the setup of the NREC COVID-19 (or perhaps foreseen for a future more general NREC)?*

The NREC COVID-19, including its bespoke Terms of Reference, was established by the Minister for Health as a temporary measure using existing laws in the system. However, a new bill is due to be enacted in 2021 – the National Research Ethics Committee Bill. This Bill, along with parallel secondary legislation on the clinical trials of medicinal products, will significantly reform the research ethics committee framework across the spectrum of health research in Ireland through the establishment of further National Research Ethics Committees with the National Office to drive and support the reforms. It is envisaged that research ethics review in Ireland will constitute a mixed-model system including both NRECs and local RECs, each with defined remits. 

- *The ‘learning experiences’ seems to have been positive, and few mistakes or miscalculations seem to have been made. This is commendable, but would in hindsight perhaps other decisions have been made that help other countries?*

We’ve attempted to capture the ways in which the process could have been improved in hindsight in the publication within the suggested word limits. If you have any suggestions of areas that we may have missed, we would be happy to consider these.

- *How did (or will) the experience with the NREC COVID-19 influence ideas about the design of a centralized ethics and regulatory review system in the future (in Ireland, but perhaps also in a European perspective, considering the increasing influence of European regulations like European Clinical Trial Regulation (EU 536/2014), Medical Device Regulation (EU 2017/745) and In Vitro Diagnostics Regulation (EU 2017/746))?*

The experience of the NREC COVID-19 will act as a proof of principle for the delivery and management of future National RECs. The next National RECs are likely to be in response to the Clinical Trial Regulation and the Medical Device Regulation.

*(It probably remains to be seen which scope will be chosen for a ‘general NREC’a in the future, outside of an epidemic. The Central Review Decree of the Dutch Central Committee on Research Involving Human Subjects (CCMO) could serve as an example ( https://english.ccmo.nl/investigators/legal-framework-for-medical-scientific-research/decrees-and-ministerial-regulations/central-review-of-medical-research-involving-human-subjects-decree-bcb).)*

 This is very helpful.

# Peer review: Reviewer response for version 1

**DOI:** 10.21956/hrbopenres.14260.r27961

**Published:** 2020-09-30

**Authors:** Rachel K. Crowley

**Affiliations:** 1 Department of Endocrinology, St Vincent's University Hospital, Dublin, Ireland; 2 University College Dublin, Dublin, Ireland

The authors have provided a description of the requirement and delivery of a National Research Ethics Committee opinion on studies of COVID-19. Reporting this experience is valuable for 2 reasons; 1) COVID-19 challenged the ethical review of research generally, so reporting experience in this context is of wide value, and 2) this was the first experience of a national REC in Ireland. The authors and committee should be commended for their work in both delivering review of 93 studies and taking the time to share the experience.

 The letter would benefit from more detail in order to maximise its usefulness to international readers. I think some small changes to provide further detail and a few clarifications are all that are required.

 For example -

1. p5 - how were the information technology projects chosen to support the committee? The authors may not wish to name them, but the criteria for selection and experience of same would be worth reporting.
2. p5 - consider briefly describing the complex ethical considerations mentioned. Were they COVID-specific? Or due to the challenges of a new national REC process?
3. p5 - how was feedback provided to the research community? Did this change processes?
4. How were REC members selected and recruited?
5. p5 - how was the HRB support leveraged, and was the HRB funding any COVID-19 studies that could be considered a conflict of interest?

Clarification

1. 93 projects were reviewed - were more submitted but declined for review because of the revised prioritisation process?
2. It appears that some studies were not returned for review after provisional opinion, is there detail on why this was so?
3. Suggest clarify in the manuscript that 12 RECs in Ireland approved to give a single national approval to CTIMPs and that local RECs did continue to operate in COVID-19 times.

Suggest cite the WHO/RFH/20.1 ethical standards for research during public health emergencies guidance updated for COVID.

Does the article adequately reference differing views and opinions?

Partly

Are all factual statements correct, and are statements and arguments made adequately supported by citations?

Yes

Is the Open Letter written in accessible language?

Yes

Where applicable, are recommendations and next steps explained clearly for others to follow?

Partly

Is the rationale for the Open Letter provided in sufficient detail?

Yes

Reviewer Expertise:

Endocrinology. Research Governance.

I confirm that I have read this submission and believe that I have an appropriate level of expertise to confirm that it is of an acceptable scientific standard, however I have significant reservations, as outlined above.

# Response

**Published:** 2020-11-03

**Authors:** Aileen Sheehy

**Affiliations:** Health Research Board, Ireland

Dear Prof. Crowley,

 Thank you for taking time to review our work, which will help us improve this Open Letter. We have revised the publication, informed by your comments and those of the second reviewer. We have also replied to your comments and queries directly below:

*1. How were the information technology projects chosen to support the committee? The authors may not wish to name them, but the criteria for selection and experience of same would be worth reporting.*

 The tools for video conferencing and an online reading room were selected based on the security and functionality afforded, in addition to ease of use. The selection was guided by the expertise of our local IT support team. Another national committee of similar size and comparable remit had a positive experience with the particular online reading room tool selected.

 This tool met the needs of the NREC COVID-19 – specifically, the safe and secure upload of applicant documentation, functionality to record comments, and easy to navigate. Members were given a step-by-step orientation on both the video conferencing and online reading room tools and had access to IT support on request. To our knowledge, no member had prior experience of the online reading room tool and few members had prior experience of the video conferencing tool.

*2. Consider briefly describing the complex ethical considerations mentioned. Were they COVID-specific? Or due to the challenges of a new national REC process?*

 Although there were many new challenges setting up a national REC process, the emphasis of the complexity lay in the ethical considerations, which were primarily COVID-specific or particular to a public health emergency. Many of prospective participants for COVID research studies may be incapacitated and a consent declaration required; therefore, one complex consideration was the justification for, and best practice in, capturing informed assent from people close to the participant such as family members, partners or friends.

 In terms of enabling informed consent, e-consent and telephone consent was used frequently in the research studies under review due to the social restrictions in place. The potential implications for these approaches were discussed by the Committee.

 Due to the prevailing social restrictions, new digital communication and research platforms were used in COVID-related studies. The implications of using these platforms for the protection of personal data of participants was another complex consideration. While mindful of the imperative to share knowledge in a timely manner, the Committee frequently sought evidence and reassurances that participants’ personal information would be protected throughout the research study when managed by any digital tools, including within international collaborations.

  We will ensure they are adequately captured in the revised version of the publication.

*3. How was feedback provided to the research community? Did this change processes?*

 Feedback from the NREC COVID-19 came in the form of decision letters provided to applicants by the National Office on behalf of the Committee; most decision letters relayed decisions of provisional approval. The queries, conditions and recommendations underpinning the provisional approval letters were actionable and were provided to strengthen the research studies from an ethical perspective. Where common issues arose, the associated feedback was standardised where possible across applications with a view to informing best practice.

 Senior staff at the National Office did provide advice and guidance on occasion to researchers on their applications in advance of consideration by the NREC COVID-19, where there were clear administrative improvements to be made or where the application would benefit from additional supporting information.

 This process did not change for the duration of the NREC COVID-19.

*4. How were REC members selected and recruited?*

 Members of the NREC COVID-19 were formally appointed by the Minister of Health. Members were selected based on their knowledge, expertise, and previous experience on local research ethics committees, with due regard for the diversity of skills and experience necessary to enable an informed review of the applications likely to come under review. In line with international best practice, the NREC COVID-19 benefitted particularly from the contributions of those members representative of PPI.

 As the NREC COVID-19 was set up as a matter of urgency, time did not allow for formal training of members in research ethics. For this reason, many of the members were selected based on their experience sitting on research ethics committees (RECs) or similar types of committees.

 We will add some additional information on this in the paper.

 5. *How was the HRB support leveraged, and was the HRB funding any COVID-19 studies that could be considered a conflict of interest?*

 The National Office for Research Ethics Committees is an independent office with a statutory function, housed within the HRB. The role of the HRB is restricted to operational and infrastructural support for the National Office team. During health emergency, this encompassed website space, and IT support; this was critical to display information on the Committee, the application process, and minutes and decisions from meetings to ensure transparency in the process. The National Office also availed of the HRB communications expertise to leverage established communication channels to publicise the launch of the NREC COVID-19, and any updates or developments to the wider Irish research community.

 The HRB funded COVID-19 research studies through the Rapid Response Funding call, a number of which were reviewed by the NREC COVID-19. These applications proceeded through the standard NREC review process and applicants liaised with the Committee solely through the National Office team. Committee members were asked to declare any conflicts at the start of every meeting and decisions were uniformly made by consensus. Senior National Office staff acted as observers during NREC meetings and did not contribute to decisions on any application. Discussions on all applications reviewed by the NREC COVID-19 are detailed in the meeting minutes, which are publicly available.

 HRB unequivocally had no role in the decision-making process of the NREC COVID-19, nor will it have for future NRECs; this is provided for in the General Scheme of the National Research Ethics Committees Bill (July 2019).

 We will add more clarity around this in the paper to ensure there is no perception of a potential conflict of interest.

**Clarifications**

 1. *93 projects were reviewed - were more submitted but declined for review because of the revised prioritisation process?*

 For workflow management purposes and Committee capacity, it was necessary to prioritise the scope of research for review. Several applications were submitted to the NREC COVID-19 that did not fit within the prioritised scope. For these studies, the National Office suggested that applicants may be better served using their local REC; in most instances, this was the option chosen by the applicant. In a few instances, the applicant put forward strong justification as to why their study should be reviewed by the NREC COVID-19 rather than a local REC. For these, the Chair decided whether the applications would be reviewed by the NREC COVID-19.

 2. *It appears that some studies were not returned for review after provisional opinion, is there detail on why this was so?*

 Out of the 93 applications reviewed, 83 applications received provisional approval. Out of the remaining 10 applications, 4 were declined, 5 received full approval outright, 1 application was reviewed but was missing a core document so a decision could not be made.

 When this paper was first submitted, 81 final approvals were issued. This figure has increased to 85 approvals. Two applicants notified the National Office at provisional approval stage that their studies will not be proceeding, and one applicant has had their provisional approval revoked due to lack of response.

 We will add the revised numbers to the next iteration of the paper.

 3. *Suggest clarify in the manuscript that 12 RECs in Ireland approved to give a single national approval to CTIMPs and that local RECs did continue to operate in COVID-19 times.*

 This is an important point and we are happy to make this clearer in the next version of the publication.

*Suggest cite the WHO/RFH/20.1 ethical standards for research during public health emergencies guidance updated for COVID.*

 We are happy to add this additional citation as this is an important document that did help to inform our processes.
